# P-2029. Improving Viral Hepatitis Testing with a Clinical Scenario Based Order Set: The Viral Hepatitis Hub

**DOI:** 10.1093/ofid/ofaf695.2193

**Published:** 2026-01-11

**Authors:** Jordan Feingold-Link, Courtney E Comar, Jonathan Fenkel, Kory S London, Sean Moss, Elizabeth Novick, Christine Papastamelos, Matthew Pettengill, Jeffrey Riggio, Daniel Taupin

**Affiliations:** Thomas Jefferson University Hospital, Philadelphia, PA; Thomas Jefferson University, Philadelphia, Pennsylvania; Thomas Jefferson University Hospital, Philadelphia, PA; Thomas Jefferson University, Philadelphia, Pennsylvania; Thomas Jefferson University Hospital, Philadelphia, PA; Thomas Jefferson University Hospital, Philadelphia, PA; Thomas Jefferson University Hospital, Philadelphia, PA; Department of Clinical Laboratories, Thomas Jefferson University Hospitals, Philadelphia, PA; Jefferson Health, Philadelphia, Pennsylvania; Thomas Jefferson University Hospital, Philadelphia, PA

## Abstract

**Background:**

Viral hepatitis (VH) screening is often suboptimal, with historically marginalized populations facing significant disparities in both testing and treatment. Paradoxically, providers frequently order inappropriate hepatitis tests, particularly acute-phase markers like HAV IgM and HBV core IgM in clinical scenarios where they aren’t indicated. Our specific aim was to improve local VH testing appropriateness and reduce wasteful ordering by implementing a clinical scenario-based electronic health record (EHR) order set.

**Figure:**
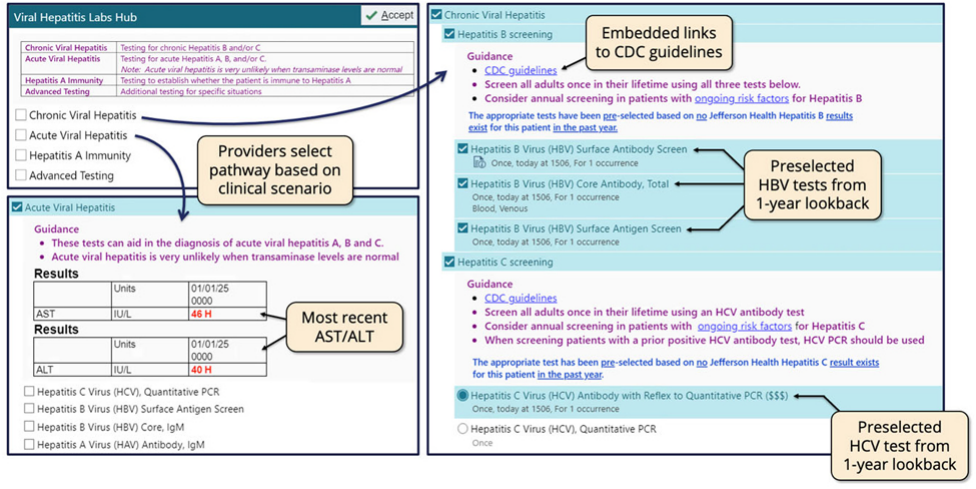
Order Set Features

**Figure:**
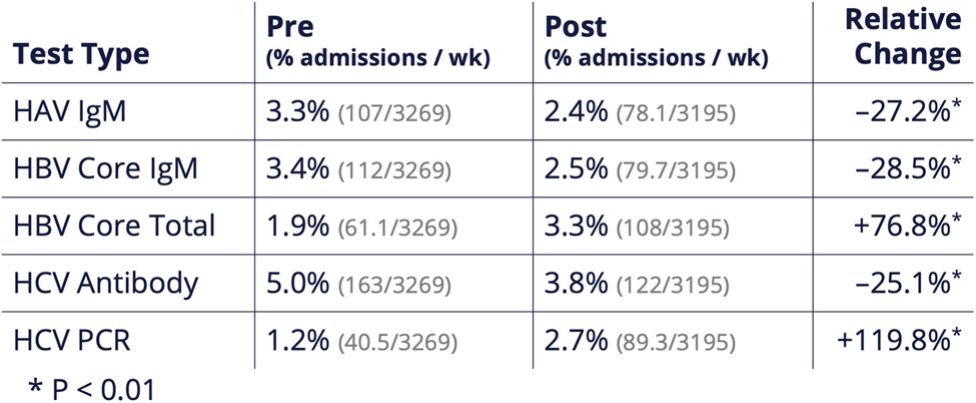
Overall Test Volume Change

**Methods:**

We created an electronic order set for VH testing organized by clinical scenario rather than by virus type, to better align with clinical decision-making and evidence-based guidelines. [FIGURE 1] It featured automated clinical decision support (CDS) including prior result checking, lab displays, and links to guidelines.

We adopted a pre-post analysis (24 weeks pre, 18 weeks post) using EHR data to compare VH test rates and appropriateness. Appropriateness measures included acute marker orders (HAV/HBV IgM) in patients without elevated alanine aminotransferase (ALT), and repeat HCV Ab tests within 1 year of a prior positive. Changes were assessed using Chi-squared significance testing (p < 0.001) and Statistical Process Control (SPC) charts.

**Figure:**
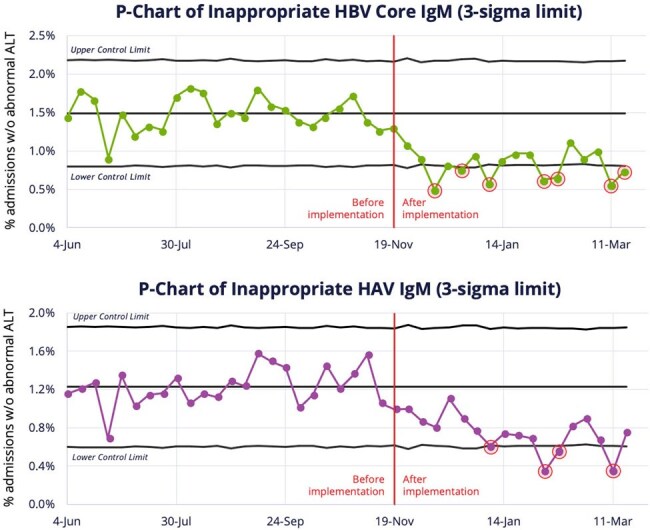
P-Charts of Inappropriate HAV IgM and HBV Core IgM

**Results:**

Post-intervention (all p < 0.001): Orders decreased for HAV IgM (down 27.2%) and HBV Core IgM (down 28.5%), while increasing for HBV Core Total Ab (up 76.8%) and HCV PCR (up 119.8%). [FIGURE 2] Appropriateness improved: inappropriate repeat HCV Ab testing fell 34.6%. Among patients without elevated ALT, inappropriate HAV IgM orders dropped 37.4% and HBV Core IgM orders dropped 41.8%. SPC charts demonstrated special cause variation, confirming a reduction in inappropriate testing. [FIGURE 3]

**Conclusion:**

The scenario-based order set with CDS improved VH test appropriateness and resource use by reducing unnecessary tests and aligning orders with guidelines. This EHR strategy offers a replicable model for improving lab utilization. Next steps involve refining CDS and extending screening pathways for high-risk groups.

**Disclosures:**

Jonathan Fenkel, MD, Alexion: Research Support to Institution|Bristol-Myers Squibb: Advisor/Consultant|Gilead: Advisor/Consultant|Gilead: Research Support to Institution|Ipsen: Research Support to Institution

